# Effects of Tyrosine and Tryptophan in Rats with Diet-Induced Obesity

**DOI:** 10.3390/ijms22052429

**Published:** 2021-02-28

**Authors:** Vladimir A. Shipelin, Nikita V. Trusov, Sergey A. Apryatin, Antonina A. Shumakova, Anastasia S. Balakina, Nikolay A. Riger, Ivan V. Gmoshinski, Dmitry B. Nikityuk

**Affiliations:** 1 Federal Research Centre of Nutrition and Biotechnology, 109240 Moscow, Russia; nikkitosu@yandex.ru (N.V.T.); apryatin@mail.ru (S.A.A.); antonina_sh@list.ru (A.A.S.); balakina.a.s@yandex.ru (A.S.B.); riger@ion.ru (N.A.R.); gmosh@ion.ru (I.V.G.); dimitrynik@mail.ru (D.B.N.); 2 Academic Department of Innovational Materials and Technologies Chemistry, Plekhanov Russian University of Economics, 115093 Moscow, Russia; 3 Department of Operative Surgery and Topographic Anatomy, I.M. Sechenov First Moscow State Medical University (Sechenov University), 119991 Moscow, Russia

**Keywords:** tyrosine, tryptophan, obesity, rats, behavior, memory, cytokines, inflammation, liver morphology

## Abstract

Amino acids tyrosine (Tyr) and tryptophan (Trp) play a significant role in the regulation of energy metabolism, locomotor activity, and eating behavior. We studied the possibility of modulating these processes in obesity by increasing the pool of Tyr and Trp in the experimental diet. As a model of obesity, we used Wistar rats fed a diet with an excess specific energy value (HFCD) for 64 days. Trp led to a normalization of the rats’ body weight almost to the control level, but increased anxiety-like behavior and decreased long-term memory. The consumption of amino acids resulted in increased grip strength and impairment of short-term memory. The locomotor activity of animals decreased with age as a result of Tyr consumption, while Trp, on the contrary, prevented this. The Tyr supplementation led to the normalization of triglycerides and LDL. In the spleen cell lysates, amino acids suppressed the production of proinflammatory cytokines. The liver tissue morphology showed that the consumption of Tyr noticeably weakened the signs of fatty degeneration. The addition of Trp, on the contrary, led to an unfavorable effect, consisting of the appearance of a high number of large rounded fatty vacuoles. The data obtained indicate a more pronounced anti-inflammatory effect of Tyr as compared to Trp.

## 1. Introduction

Modern approaches to obesity diet therapy are based on the ability of nutrients to influence actively the level of physical and metabolic activity, the balance of fat accumulation and utilization, and the intensity of inflammation in adipose tissue [1]. In this regard, it is of great interest to study the impact of dietary factors on the neuroendocrine regulation of energy metabolism processes, mediating eating behavior [2,3], hunger, and appetite [4].

The neurotransmitters dopamine [5], serotonin [6], and their minor metabolites (trace amines, etc.) [7] play a critical role in the regulation of energy metabolism, locomotor activity, and eating behavior. The metabolic precursors of these neurotransmitters are large neutral amino acids-tyrosine (Tyr) and tryptophan (Trp). There is an association between the changes in dopamine signaling in the striatum and locomotor activity [8]. In turn, the effect of serotonin on metabolic processes is due by the activation of the signaling pathway in the hypothalamic neurons, which are a part of the pro-opiomelanocortin system (POMC) [6]. Serotoninergic neurons receive information about the composition of the diet due to the competition of Trp, Tyr, and other large neutral amino acids (phenylalanine, leucine, isoleucine, and methionine) for transport across the blood-brain barrier [9]. An excessive intake of fat and carbohydrates with the diet leads to a decrease in the catabolism of tissue proteins containing small amounts of Trp. This contributes to an increase in the specific content of Trp in the pool of free amino acids in blood plasma and an increase in its transport to the brain, where Trp is converted into serotonin, which has anorexigenic and sedative effects.

In this regard, the question arises about the possibility of directed modulation of the processes of lipid and energy metabolism, physical activity, and eating behavior by changing the ratio of Trp and Tyr supplied with food in the total pool of dietary amino acids. The aim of this work was to study the effect of additional amounts of dietary Tyr and Trp on the behavioral responses, metabolic parameters, and levels of adipokines and cytokines in Wistar line rats fed a diet with an excessive specific energy value, which provokes the development of dietary obesity.

## 2. Results

### 2.1. Integral Indices

**Figure 1:**
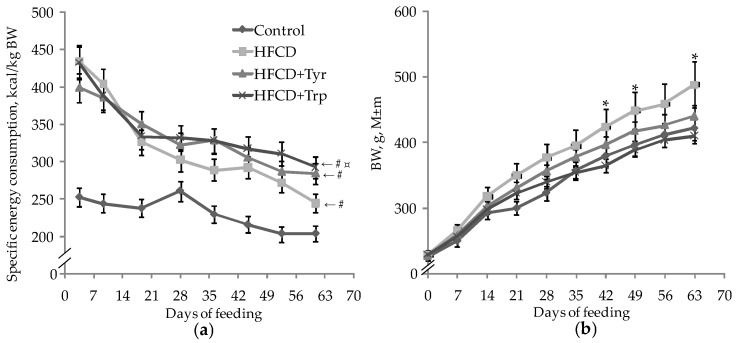
Changes in specific dietary energy consumption per kg body weight (**a**) and body weight (**b**) of rats in the control and experimental groups during the experiment. * the distribution is heterogeneous, *p <* 0.05, ANOVA by the factor “Trp”; # the difference with the control group is significant; ¤ the difference with the HFCD group is significant, *p <* 0.05, *t*-test for pairwise comparisons of group mean values. The number of animals in the groups was 8.

Throughout the experiment, the rats of all groups were characterized by a gradual decrease in energy consumption from the experimental diet, and the animals of three groups receiving HFCD consumed significantly more food energy per unit of body weight compared to the control group (*p* < 0.05, Student’s *t*-test for pairwise related mean group values) (Figure 1a). Starting from the 21st day of the experiment the addition of Trp led to a significant increase in specific energy consumption in comparison with the HFCD consumption without the additive. The rats that received only HFCD were characterized by the highest body weight gains (Figure 1b). The addition of Trp to HFCD led to a significant decrease in body weight, almost to the control level, despite the highest specific energy consumption in this group.

**Figure 2:**
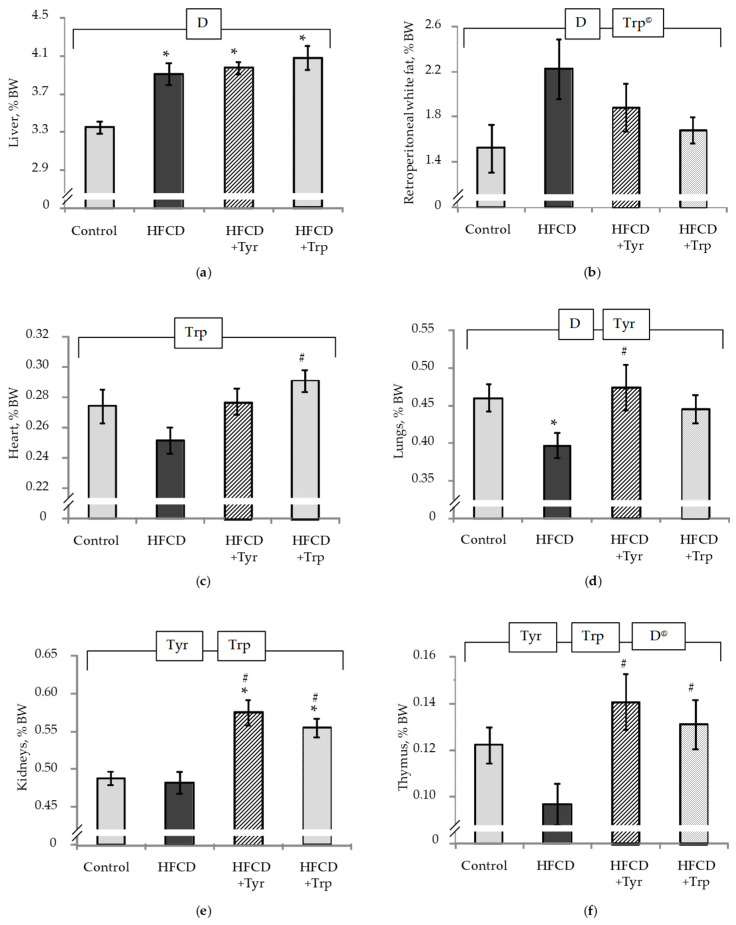
Weight indexes (%BW) of liver (**a**), retroperitoneal white fat tissue (**b**), heart (**c**), lungs (**d**), kidneys (**e**) and thymus (**f**), mean ± standard error of the mean. * the difference with the control group is significant; # the difference with the group receiving HFCD is significant, *p <* 0.05, Mann-Whitney U test. The bracket above the bars denotes significant dependence on the factors: “diet” (D), “tyrosine” (Tyr), “tryptophan” (Trp), *p <* 0.05, 3-way ANOVA. Index @-trend level difference, *p <* 0.1. The number of animals in the groups was 8.

At the end of the experiment, the rats of all groups treated with HFCD were characterized by an increased relative weight of the liver (Figure 2a) and retroperitoneal white fat (Figure 2b), (*p <* 0.05, ANOVA, according to the “diet” factor). However, in the rats treated with Trp supplementation, there was a tendency towards a decrease in white fat mass (*p <* 0.1, ANOVA, factor “Trp”). The relative heart mass (Figure 2c) in these animals was higher than in those who received the HFCD (*p <* 0.05, Mann-Whitney *U*-test) and did not differ from the control. A similar pattern for the relative lung mass (Figure 2d) was observed in the case of animals treated with Tyr (*p <* 0.05, ANOVA, according to the “Tyr” factor). The addition of both amino acids to the HFCD increased the relative weight of the kidneys (Figure 2e) and thymus (Figure 2f) (*p <* 0.05, ANOVA, according to the “Tyr” and “Trp” factors).

### 2.2. Muscle Tone, Memory Function and Behavioral Responses

**Figure 3:**
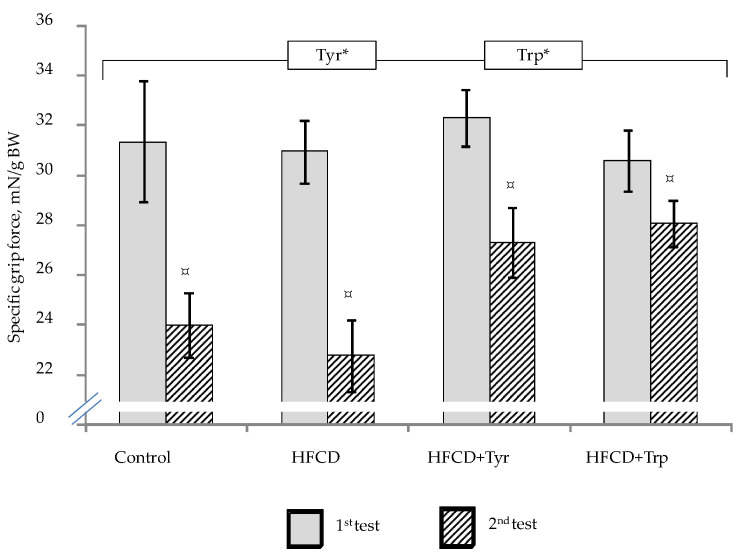
Muscle compression force (specific grip force of the forepaws) in rats during the first and second testing, mean ± standard error of the mean. The bracket above the bars denotes significant dependence on the factors: “Tyr” and “Trp”, *p <* 0.05, 3-way ANOVA. * only in the second test; ¤ the difference between the first and second tests is significant, *p <* 0.05, Wilcoxon’s criteria for pairwise comparisons. The number of animals in the groups was 8.

The muscle compression force (Figure 3) in all groups of the rats significantly decreased in the second test compared with the first (*p <* 0.05, Wilcoxon test for pairwise related values). At the same time, when comparing the groups with each other, there was no difference in the first test. In the second test, the consumption of both amino acids led to a significant increase in grip strength (*p <* 0.05, ANOVA, according to the “Tyr” and “Trp” factors for the second test).

During the first study in the CRPA test, all rats visited the dark compartment of the apparatus, where they received a not dangerous to their health electric shock to develop a conditioned passive avoidance reflex. The average latency before the first entry into the dark compartment did not differ significantly between the control, HFCD, and HFCD with Tyr. In animals treated with Trp, the mean latency was significantly reduced (*p <* 0.05, Mann-Whitney *U*-test), which may indicate both an increase in anxiety and search activity in this group (Table 1). The retention of a short-term memory trace (determined by the number of animals that did not enter into the dark compartment during the second test) did not significantly differ in rats fed the control diet and the HFCD, but it was significantly and multiple times reduced when both amino acids were consumed (*p <* 0.05, *χ*-square test). Long-term memory (assessed after 21 days) was reduced compared to controls in all three groups of rats fed with HFCD. It should be noted that all rats receiving Trp entered the dark compartment, thus, they did not preserve any memory trace at all.

**Figure 4:**
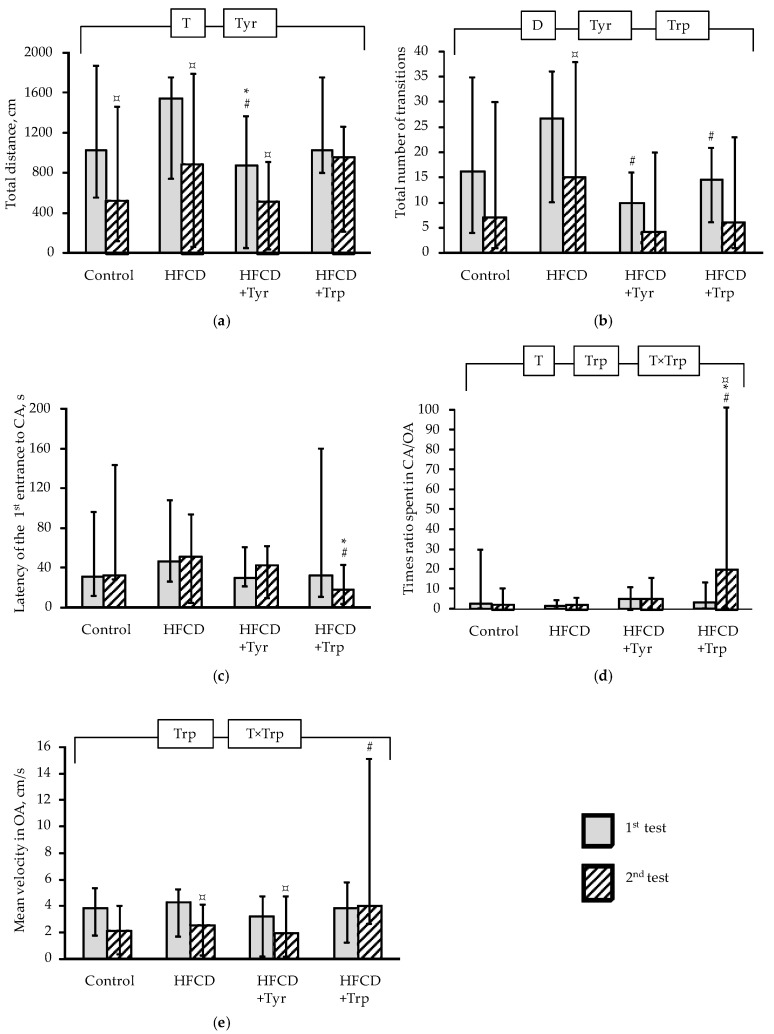
Indicators of rats tested in the EPM, (median, intervals of change): total distance traveled (**a**), total number of transitions (**b**); latency of the first entry into the closed arm (**c**); the ratio of the time spent in the closed (CA) and open (OA) arms of the maze (**d**), the average speed in the OA (**e**). * the difference with the control group is significant; # the difference with the group receiving HFCD is significant, *p <* 0.05, Mann-Whitney test; ¤ the difference with the first test is significant, the Wilcoxon test for pairwise comparisons. The bracket above the bars denotes significant dependence on the factors: “test” (T), “diet” (D), “tyrosine” (Tyr), “tryptophan” (Trp) and their combinations, *p <* 0.05, 3-way ANOVA. The number of animals in the groups was 8.

In the EPM test, the total distance covered in the maze in 180 s (Figure 4a) significantly decreased in the second test as compared to the first one, in rats receiving the control diet, HFCD and HFCD with Tyr (*p <* 0.05 Wilcoxon test for pairwise related values), but the addition of Trp canceled this effect. At the same time, the distance covered by animals receiving Tyr was the smallest among all groups, both in the first and in the second test (*p <* 0.05, ANOVA for the “Tyr” factor). From this, we can conclude that the locomotor activity of animals decreases as a result of the Tyr consumption, and the addition of Trp, on the contrary, maintained locomotor activity with age. The indicator of the total transitions number between the maze arms (Figure 4b), which characterizes the level of search activity, also decreased in the second test as compared to the first one; however, this decrease was significant only in the group that received the HFCD without supplements. Both Tyr and Trp reduced the number of transitions between the maze arms (*p <* 0.05 ANOVA for the “Tyr” and “Trp” factors), and in the first test this effect was even more pronounced than in the second one since it was also observed in a paired comparison with the group receiving the HFCD (*p <* 0.05, Mann-Whitney test). The latency before the first entry of the animal into the closed arm (CA) of the maze (Figure 4c) in rats receiving Trp supplementation was minimal in the second test among all groups and significantly reduced in comparison with the HFCD group. At the same time, in the second test of animals consuming Trp, the ratio of the times spent in the CA and the open arm (OA) of the maze (Figure 4d) and the average movement speed in the OA (Figure 4e) (*p <* 0.05, ANOVA, according to the factors “Trp” and “Test × Trp”; *p <* 0.05 compared with the HFCD group, according to the Mann-Whitney *U*-test). The last three indicators in total show an increase of anxiety-like behavior in the second test in rats receiving Trp supplementation, which is also in qualitative agreement with the data in the CRPA test.

### 2.3. Biochemical Indices

**Figure 5:**
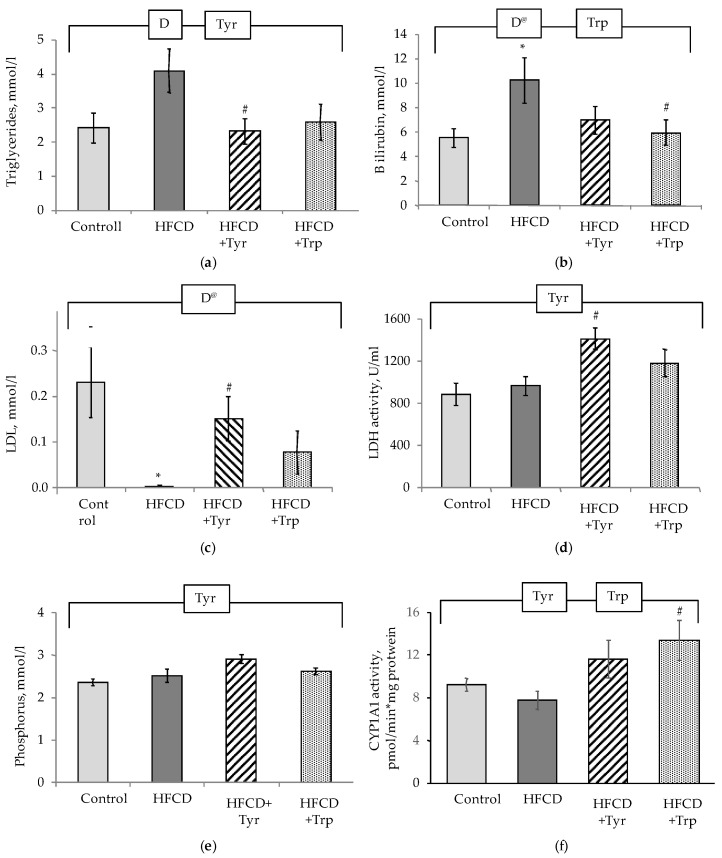
Biochemical parameters of blood plasma and liver of rats, mean ± standard error of the mean. Triglycerides (**a**), bilirubin (**b**), LDL cholesterol content (calculation) (**c**), LDH activity (**d**), phosphorus content (**e**), CYP1A1 activity in the microsomal fraction (**f**). *the difference with the control group is significant; # the difference with the group receiving HFCD is significant, *p <* 0.05, Mann-Whitney test. The bracket above the bars denotes significant dependence on the factors: “diet” (D), “tyrosine” (Tyr), “tryptophan” (Trp), *p <* 0.05, 3-way ANOVA. Index @ excluding rats treated with Tyr. The numbers of animals in the groups were 8.

In comparison with the control, the HFCD consumption by rats led to an increase of triglycerides concentration in the blood plasma (Figure 5a), total bilirubin level (Figure 5b), a decrease in the content of cholesterol in the LDL fraction (Figure 5c), their ratio with HDL, and the level of urea (data not shown) (*p <* 0.05, ANOVA, on the “diet” factor in all these cases). The addition of Tyr to the HFCD composition led to the normalization of triglycerides and LDL levels; for the bilirubin level, Trp supplementation had the same effect. In the group of rats receiving Tyr, the highest average LDH activity (Figure 5d) and the phosphorus level (Figure 5e) in the blood plasma (*p <* 0.05, ANOVA, according to the “Tyr” factor) were observed. The rest of the studied biochemical parameters did not significantly respond to the intake of HFCD and amino acid supplements by rats (data not shown). The introduction of Tyr and Trp supplements into the diet increased the activity of CYP1A1 (Figure 5f) (*p <* 0.05, ANOVA, according to the “Tyr” and “Trp” factors), while the activities of the other studied oxidoreductases and conjugating enzymes of the xenobiotic detoxification system did not respond to dietary interventions applied (data not shown).

### 2.4. Cytokines and Adipokines Levels

**Figure 6:**
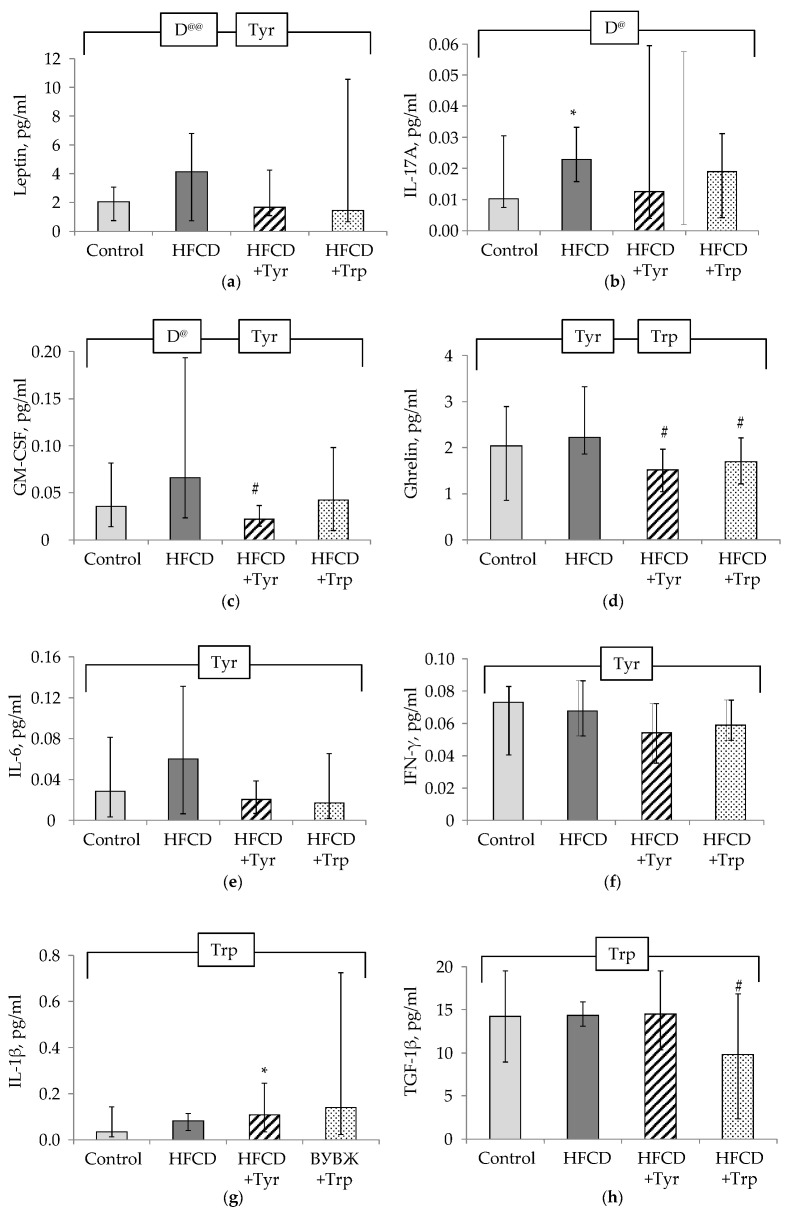
Blood plasma levels of cytokines and adipokines in the rats, median ± intervals of change. Leptin (**a**), IL-17A (**b**), GM-CSF (**c**), ghrelin (**d**), IL-6 (**e**), INF-γ (**f**), IL-1β (**g**), TGF-1β (**h**). * the difference with the control group is significant; # the difference with the group receiving HFCD is significant, *p <* 0.05, Mann-Whitney test. The bracket above the bars denotes significant dependence on the factors: “diet” (D), “tyrosine” (Tyr), “tryptophan” (Trp), *p <* 0.05, 3-way ANOVA. Indices @ excluding rats receiving Trp, @@ excluding rats receiving Tyr. The number of animals in the groups was 8.

**Figure 7:**
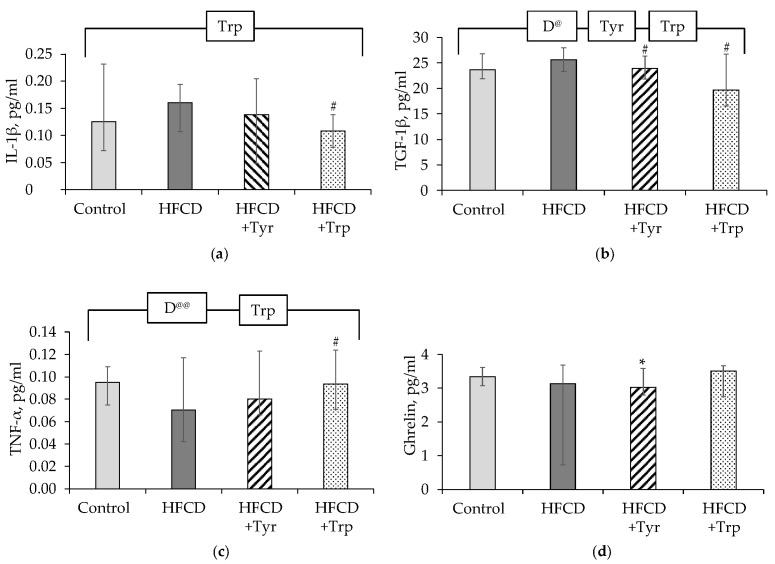
Levels of cytokines and adipokines in the lysates of white adipose tissue in the rats, median ± intervals of change: IL-1β (**a**), TGF-1β (**b**), TNF-α (**c**), ghrelin (**d**). * the difference with the control group is significant; # the difference with the group receiving HFCD is significant, *p <* 0.05, Mann-Whitney test. The bracket above the bars denotes significant dependence on the factors: “diet” (D), “tyrosine” (Tyr), “tryptophan” (Trp), *p <* 0.05, 3-way ANOVA. Indices @ excluding rats receiving Trp, @@ excluding rats receiving Tyr. The numbers of animals in the groups were 8.

In the rats consumed HFCD, compared with the control, there were increased levels in the blood plasma of leptin (Figure 6a), cytokines IL-17A (Figure 6b) and GM-CSF (Figure 6c) (*p <* 0.05, ANOVA, on the factor “diet”; in the latter case, the difference is significant without taking into account the animals receiving Tyr). The consumption of Tyr supplements by rats led to shifts in the plasma cytokine profile, which consisted of a decrease in the levels of leptin, ghrelin (Figure 6d), GM-CSF, IL-6 (Figure 6e), IFN-γ (Figure 6f), an increase in IL-1β (Figure 6g). The addition of Trp to the HFCD led to an increase in the level of IL-1β, a decrease of ghrelin, and TGF-1β (Figure 6h) (*p <* 0.05, ANOVA for the factor “Trp” in all these cases). In lysates of white adipose tissue cells, Trp supplementation led to a significant decrease in the production of IL-1β (Figure 7a), TGF-1β (Figure 7c), and an increase in TNF-α (Figure 7c) (*p <* 0.05, ANOVA, by factor “Trp”). Tyr consumption led to a small in absolute value, but a significant decrease in the level of TGF-1β and ghrelin (Figure 7d).

**Figure 8:**
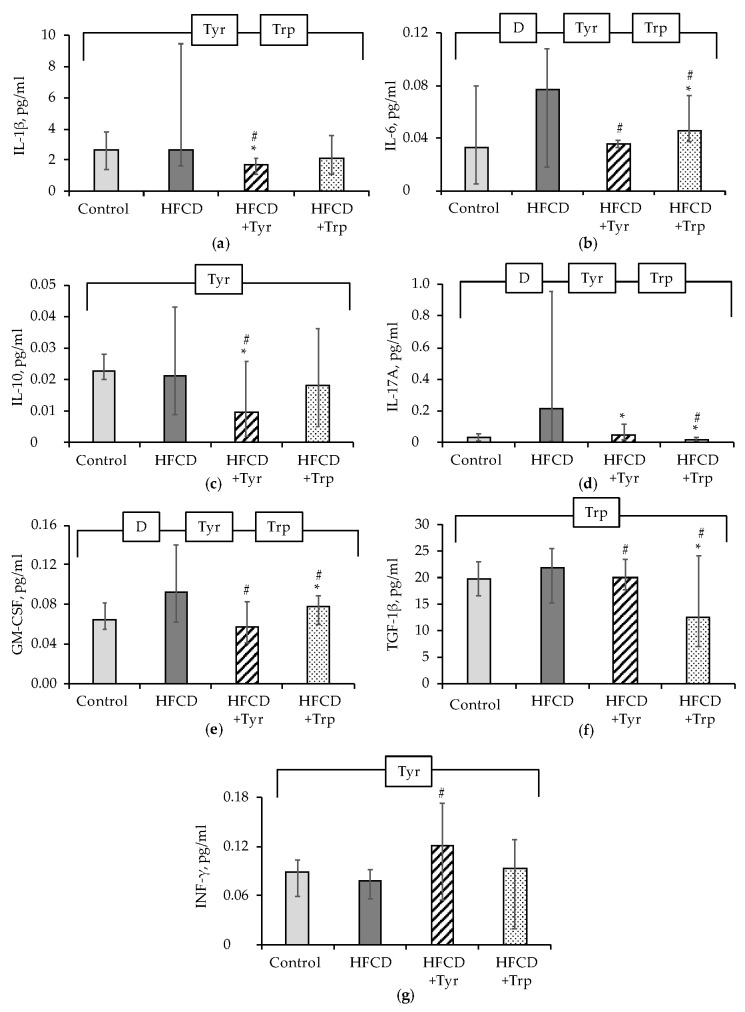
Levels of cytokines and adipokines in the rats’ spleen, median ± intervals of change: IL-1β (**a**) IL-6 (**b**) IL-10 (**c**) IL-17A (**d**) GM-CSF (**e**) TGF-1β (**f**) INF-γ (**g**). * the difference with the control group is significant; # the difference with the group receiving HFCD is significant, *p <* 0.05, Mann-Whitney test. The bracket above the bars denotes significant dependence on the factors: “diet” (D), “tyrosine” (Tyr), “tryptophan” (Trp), *p <* 0.05, 3-way ANOVA. The numbers of animals in the groups were 8.

The most contrasting effects of amino acids were observed for the content of cytokines in spleen cells lysates, where Tyr supplementation significantly suppressed the production of IL-1β (Figure 8a), IL-6 (Figure 8b), IL-10 (Figure 8c), IL-17A (Figure 8d), GM-CSF (Figure 8e), TGF-1β (Figure 8f), and increased the production of IFN-γ (Figure 8g). In rats treated with Trp, the production of IL-6, IL-17A, GM-CSF, and TGF-1β in the spleen was significantly reduced in comparison with the HFCD group.

### 2.5. Liver Histology

**Figure 9:**
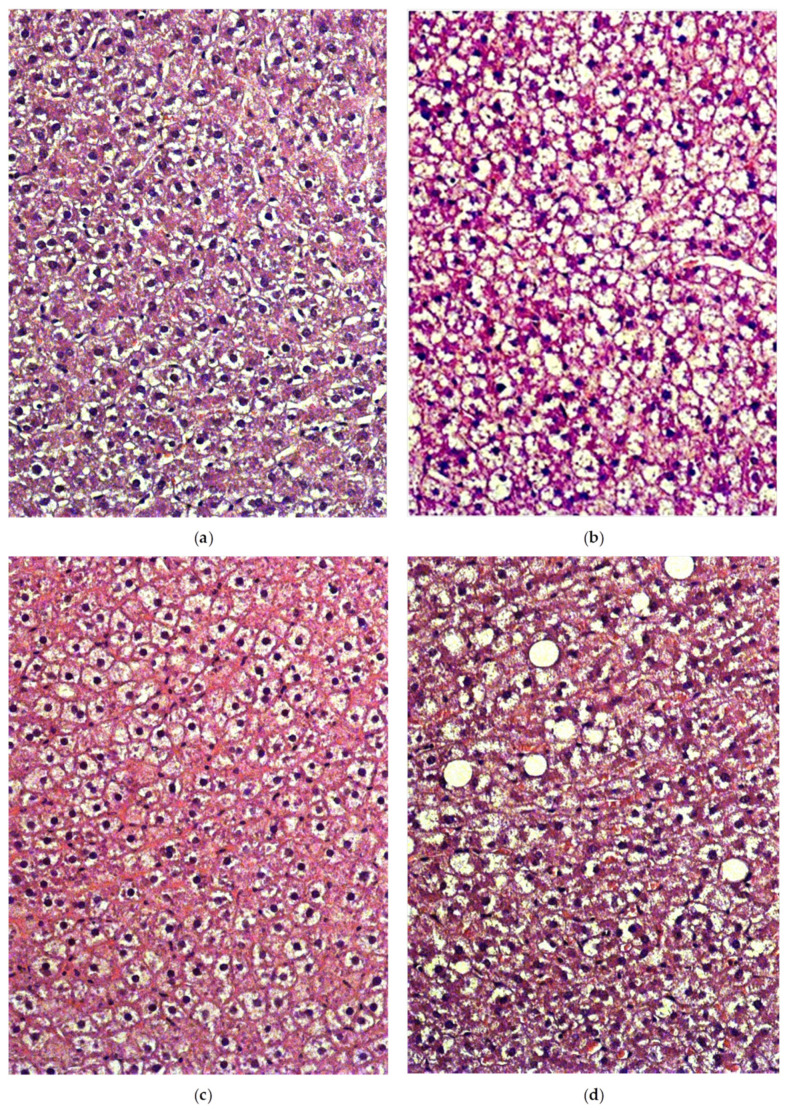
Light-optical micrographs of the liver sections of rats of the control group (**a**); HFCD (**b**), HFCD with tyrosine (**c**) and tryptophan (**d**). Hematoxylin and eosin stain. Magnification ×200. The numbers of animals in the groups were 4.

The histological study showed that the lipids accumulation was observed in the liver tissue of control rats (Figure 9a) to a certain extent. At the consumption of HFCD (Figure 9b), fatty degeneration was noticed with the appearance of a large number of cells filled with lipid vacuoles. Against the Tyr consumption (Figure 9c), the signs of fatty degeneration were not so pronounced, while the Trp supplement (Figure 9d), on the contrary, led to an unfavorable effect, consisting of the appearance of many large rounded fatty vacuoles devoid of the internal cellular structure.

## 3. Discussion

As shown by the studies, the 63-day consumption of HFCD, which had an increased energy value in comparison with the control diet, led to the development in rats of the obesity phenotype: an increase in total body weight, relative mass of retroperitoneal white fat, triglyceridemia, and leptinemia. It should bear in mind that a sharp decrease in LDL cholesterol under the influence of HFCD does not indicate a reduction of atherogenic potential in rats (in contrast to humans), but point out a weakening of the process of lipid transport from the liver to peripheral organs [10]. As a result, this led to the development of hepatic steatosis, as evidenced by an increase in relative liver weight and a histologically revealed increase of lipids accumulation in hepatocytes together with the development of hyperbilirubinemia. According to the literature, cells that predominantly accumulate fat in the liver tissue can be fat-storing stellate cells (Ito cells) expressing anillin, Ki-67, CXCL14, the IAP family of apoptosis inhibitors (c-IAP1, c-IAP2, and XIAP), and cholesterol-25-hydroxylase. The resulting activation of SREBP-1 and SREBP-2 promotes the acquisition by Ito cells of a phenotype similar to adipocytes [11].

An increase in circulating levels of cytokines (IL-1β, IL-6, and IL-17A) and their production under the influence of the HFCD diet is also specific for the development of diet-induced obesity and metabolic syndrome [12,13,14]. At the same time, an increase in the TGF-β production together with increased IL-6 causes both the differentiation of effector Th17 cells producing IL-17 and prevents the naive lymphocyte differentiation into Th1 and Th2 cells, as a result of which, probably, the production of IFN-γ decreases [15].

The introduction of Tyr and Trp supplements into the HFCD diet led to the modulation of behavioral reactions and parameters of some metabolic processes that did not have an unambiguous direction. In rats that consumed Trp, despite the highest diet energy value among other groups, there was a decrease in the rate of body weight increase, the weight of accumulated retroperitoneal white fat, and, as a result, an increase in the proportion of the heart, kidneys, and thymus in the total body weight. Apparently, these effects are a consequence of increased energy expenditure due to the raised locomotor activity of these animals. Indeed, as shown by studies in EPM, Trp supplementation canceled the decrease in locomotor activity in the second test (with increasing age of the animals), in contrast to all other groups, including the control. Besides, Trp induced changes that can be interpreted as anxiety-like behavior (an increase in the ratio of the CA/OA times) or as an increase in search activity (a decrease in the latency time for entering in CA of the EPM and CRPA dark compartment; an increase in the average movement speed in the OA). Trp caused deterioration in short-term memory in the CRPA test. The decrease in long-term memory in these animals was, apparently, the most pronounced among all groups receiving HFCD.

The ability of Trp supplementation to enhance anxiety or to increase the search activity of rats, in combination with an increase in muscle tone, conflicts with the postulated sedative effect for Trp (as a precursor of serotonin) [16]. Earlier, we observed a similar Trp effect on behavioral responses in the studies on DBCB tetrahybrid mice, characterized by a high allelic diversity of the genome, similar to that of the outbred rat line (data currently in print). The reasons for the paradoxical nature of the Trp influence on behavioral reactions should be sought, as shown by a literary search, in the sphere of the interaction of this amino acid with the intestinal microbiome. As is known, indole and indolyl-3-propionic acid are among the main microbial metabolites of Trp [17]. The first one, entering the liver, is transformed there under the action of microsomal monooxygenases and sulfatases into indoxyl sulfate, which has a toxic and prooxidant effect [18]. The second metabolite, on the contrary, is regarded as a trap for free radicals and can exert various organoprotective effects under conditions of oxidative stress [19]. The ratio in the activity of different microbiota populations, alternatively synthesizing these metabolites, can vary within broad limits [20], and under certain conditions, the production of a potentially neurotoxic metabolite can prevail. Excessive formation of indole oxy derivatives in the liver can also be enhanced by the feedback mechanism due to the observed induction of the isoform of microsomal monooxygenase CYP1A1 in the liver, through the characteristic stimulation of nuclear aryl hydrocarbon receptor (AhR) for indole derivatives [21,22].

The neurotropic effect of Tyr supplementation in rats was manifested in the cancellation of a decrease in the number of transitions between arms with age in rats (see Figure 4b) and impairment of short-term memory. Similar effects were found in experiments on DATKO knockout rats characterized by excessive accumulation of secondary dopamine metabolites in the striatum of the brain [23,24]. The stimulating effect of Tyr on muscle contraction force can also be correlated with its ability to modulate dopaminergic innervation by analogy with how it occurs in animals knockout for the dopamine transporter gene [23,25]. An increase in the level of phosphorus and LDH activity in the blood plasma indicates the ability of Tyr to potentiate the processes of energy metabolism in the muscles. LDH can be considered as a marker of the intensification fatty acids beta-oxidation manifesting in an increase in the acetyl-CoA/CoA ratio in the mitochondria of myocytes and an increase in the level of pyruvate in the cytoplasm [26].

The study of the metabolic and hepatotropic effects of Tyr and Trp in rats against the background of the HFCD consumption led to ambiguous results. Tyr was characterized by an increase in the LDH level in combination with a decrease in the severity of fatty hepatosis, which indicates the ability of this amino acid to prevent excessive lipids accumulation in the liver. Similar effects were noted by us earlier in tetrahybrid DBCB mice receiving Tyr (data in print), as well as in rats with a knockout of the dopamine transporter gene (DATKO) [23]. It is known that dopamine plays an essential role in the regulation of lipogenesis due to central effects mediated by efferent dopaminergic neurons of the central nervous system [5,27,28]. In light of this, our data seem to indicate the ability of Tyr supplementation to modulate dopamine metabolism.

The decrease in the bilirubin level observed upon the addition of Trp to the HFCD can be related to the enhancement of microsomal oxidation processes in the liver under this amino acid action. On the other hand, the effect of Trp on lipid metabolism was significantly less pronounced than in the case of Tyr. However, the accumulation of lipids in the liver of rats treated with Trp apparently increased, judging by the data of the histological observation. Previously we have shown that the impact of Trp on lipid accumulation in the liver in DBCB tetrahybrid mice had a similar direction. The ability of Trp to inhibit the removal of lipids from the liver of animals consuming HFCD can be correlated both with the hepatotoxic effect of its hydroxyindole metabolites and with the supposed competition between Trp and Tyr for penetration through the blood-brain barrier, which may result in a decrease in dopamine production and weakening of its anti-obesogenic action.

The effect of both studied amino acids on the leptin and ghrelin in rats consisted in the inhibition of their production, as a result of which the leptin/ghrelin ratio (one of the markers of lipogenesis [29]) did not change significantly. These data suggest that the effects of amino acids observed in rats apparently are not mediated by the processes of orexin-mediated competitive regulation of hunger and eating behavior by leptin and ghrelin [30,31], in contrast to what we observed earlier in a similar mouse model.

According to the latest data, the development of systemic inflammation plays an important role in the pathogenesis of obesity and related conditions, such as insulin resistance and metabolic syndrome [32]. In this regard, the obtained data on the effect of the amino acids Tyr and Trp on the circulating levels and production of cytokines, which are markers of inflammatory processes in the liver and adipose tissue, are of interest. Tyr’s ability to suppress the production of cytokines IL-1β, IL-6, IL-10, IL-17A, GM-CSF and stimulate the synthesis of INF-γ was most noticeable in spleen cell lysates. Whereas the effect of Tyr on their plasma levels was less noticeable, possibly due to the high lability of cytokines in the circulation. The effect of Trp on the levels of IL-1β, IL-6, and IL-17A in rats that consumed HFCD was similar, but no significant increase in INF-γ production was observed. At the same time, an increase in the production of pro-inflammatory TNF-α playing a central role in the JNK1-mediated development of inflammation and insulin resistance of adipose tissue was observed in the white fat of rats treated with Trp [33]. The data obtained indicate a more pronounced anti-inflammatory effect of Tyr, as compared to Trp, in the model of rats consuming HFCD. One of the reasons for this difference may be the effect on the immune system of the animal organism of the Trp toxic metabolites formed in the liver. Based on the data obtained, it is very reasonable to study these amino acids in lower dose ranges in order to exclude adverse effects. It is also advisable to use complexes of amino acids or alternative delivery/encapsulation methods (in the case of Trp) to reduce the formation of its toxic derivatives.

## 4. Materials and Methods

### 4.1. Animals and Experimental Design

We used 32 male rats (age eight weeks, initial body weight 200 g) of the outbred Wistar line purchased from the Stolbovaya breeding nursery (Scientific Center for Biomedical Technologies, FMBA, Moscow region, Russia). The work with animals was performed by the Order of the Ministry of Health of the Russian Federation No. 199n of 04/01/2016 “On approval of the rules of good laboratory practice” and adhered to the standard principles described in “Guide for the Care and Use of Laboratory Animals” (8th edition). The experimental design was approved by the Ethics Committee of the Federal Research Centre of Nutrition and Biotechnology (protocol No. 4 of 04/20/2017).

The rats were divided into four groups with an equal number of eight individuals. The average body weight in the formed groups did not initially differ significantly (*p >* 0.1; ANOVA). Animals of the 1st (control) group received a balanced semi-synthetic diet according to AIN93M with some modifications [23] and drinking water purified by reverse osmosis. The 2nd group received a high-fat-carbohydrate diet (HFCD) similar in composition to the control diet with substitution of a starch part with fat (sunflower oil:lard in ratio 1:1), which made in total 30% fat by dry diet weight, and replacement of drinking water with 20% fructose solution instead of water. The 3rd group received HFCD with the addition of Tyr at a calculated dose of 1250 mg/kg of body weight (bw); 4th group—HFCD with the addition of Trp at a calculated dose of 250 mg/kg bw. The amino acids Tyr and Trp were obtained from Wirud (Germany) and were more than 99.5% pure by HPLC.

The rats were kept in pairs in polycarbonate cages at a temperature of 21 ± 1 °C under 12/12 h illumination conditions. The total duration of feeding with experimental diets was 64 days. During the experiment, the amount of food consumed and the amount of liquid consumed was determined daily, the body weight of the animals was measured weekly with an accuracy of ±0.1 g, and the appearance, activity, state of the coat, and behavioral features were monitored. Doses of amino acids consumed daily were calculated based on the weight of the diets eaten by animals. If necessary, the amounts of amino acids added to the feed were adjusted.

### 4.2. Assessment of Behavioral Responses, Memory Function and Muscle Tone Indices

The muscle tone state of the rats was evaluated by determining the front paws grip strength on the 3rd and 58th days. The grip strength was determined in mN by measuring the maximum dynamometer (Bioseb, USA) readings from two repetitions (at the moment when the animal is uncoupled from the frame).

Testing behavioral responses in the “elevated plus maze test” (EPM) was made on the 8th and 59th days. Assessing the level of anxiety-like behavior and the state of short-term and long-term memory in the conditioned passive avoidance response test (CRPA) was evaluated on the 39th, 40th, and 60th days of the experiment. Behavioral responses studies were carried out on the equipment of the Panlab Harvard Apparatus (Spain) using the methods and parameters fully described earlier [34].

### 4.3. Assessment of Integral and Biochemical Indices

Animals were euthanized on day 64 by exsanguination from the inferior vena cava under ether anesthesia. Blood was collected in volumetric tubes with 0.4 cm3 of 1% heparin solution in 0.15 M NaCl, individually recording the dilution of each sample. The mass of organs and tissues (liver, spleen, heart, thymus, retroperitoneal white fat) was determined with an accuracy of ±0.01 g. Two liver tissue samples were taken, the first of which was fixed in a solution of 3.7% formaldehyde in 0.1 M sodium phosphate buffer pH 7.00 ±0.05, dehydrated in alcohols of ascending concentration, impregnated with xylene and embedded in homogenized paraffin medium “Histomix” (BioVitrum, Moscow, Russia). Paraffin sections with a thickness of 3–4 μm were made on a Microm HM355s microtome (Leica, Germany), stained with hematoxylin and eosin according to a standard technique, and examined with an AxioImager Zl microscope (Zeiss, Germany) with a digital camera at a magnification of ×200. The second sample of liver tissue was homogenized at 0–2 °C in 0.1 M Tris-HCl buffer pH 7.4, in a ratio of 1:4 by weight. The cytosolic and microsomal liver fractions were isolated from the second liver tissue sample according to [35]. For this, the liver was homogenized at 0–2 °C in 0.1 M Tris-HCl buffer pH 7.4 in a ratio of 1:4 by weight and centrifuged in a Beckman L7-65 preparative ultracentrifuge (Beckman, USA). In both fractions, the activities of heme oxygenase-1, NAD(P)H-quinone oxidoreductase, total glutathione-S-transferase, UDP-glucuronosyltransferase, monooxygenase of the CYP1A1 isoform were determined using specific substrates according to the procedures [36,37,38,39,40].

The content in the blood plasma of glucose, triglycerides, total cholesterol and in the composition of high and low-density lipoproteins (HDL and LDL), bilirubin, urea, the total protein, albumin, the activity of alanine (ALT) and aspartate (AST) aminotransferases, lactate dehydrogenase (LDH), alkaline phosphatase, β-glutamyltransferase, calcium, phosphorus were determined on a biochemical analyzer “Konelab 20i” (Finland) according to standard methods [23].

### 4.4. Cytokines and Adipokines Analysis

Lysates of the spleen and white adipose cells were obtained in 0.1 M Tris-HCl buffer, pH 7.4, cooled to 0–2 °C, in a ratio of 1:4 by weight. The levels of cytokines GM-CSF, TGF-1β, IL-1β, IL-6, IL-10, IL-17A, INF-γ, ΤΝF-α, ghrelin, and leptin in the blood plasma and tissue lysates were determined by multiplex immunoassay using a baseline Bio-Plex Pro™ Reagent Kit V, supplemented with Bio-Plex Pro™ reagents (Rat Cytokine GM-CSF Set, Cytokine TGF-1β Set, Rat Cytokine IL-1β Set, Rat Cytokine IL-6 Set, Rat Cytokine IL-10 Set, Rat Cytokine IL-17A Set, Rat Cytokine INF-γ Set, Rat Cytokine ΤΝF-α Set, Rat Diabetes Ghrelin Set, and Rat Diabetes Leptin Set) from Bio-Rad Laboratories, Inc. (USA) on a Luminex 200 analyzer (Luminex Corporation, Austin, TX, USA) using xMAP technology using Luminex xPONENT Version 3.1 software.

### 4.5. Statistical Analysis and Data Availability

Statistical processing of the data was performed using a two-sided Student’s *t*-test for pairwise related values, three-way ANOVA, Wilcoxon-Mann-Whitney nonparametric tests as post hoc tests. The significance of the difference in proportional indicators was checked according to the Fisher’s exact test. The calculations were performed using the statistical package of SPSS^®^ 23.0 and Microsoft Excel for Windows. Differences were considered significant with the probability of accepting the null hypothesis *p* < 0.05.

## 5. Conclusions

Taken together, the data obtained suggest that the response of behavioral reactions, metabolic and immune parameters of the rat organism to the intake of additional amounts of Tyr with food is based on the ability of this amino acid to influence dopamine metabolism in the central nervous system and, presumably, in peripheral tissues. The nature of the effect of Trp on the body depends on more complex factors, in which, along with its modulating effect on the metabolism of serotonin and (indirectly) dopamine, the biological effect of toxic oxyindole metabolites formed under the action of intestinal microflora and the liver oxidoreductase system can make an important contribution. It is important to take these data into account when assessing the possible effects of high-protein Trp-rich foods on patients with obesity and metabolic syndrome, considering their possible individual metabolomic and microbiome characteristics.

## Figures and Tables

**Table 1:** Behavioral reactions of rats in the CRPA test.

Group	Rats Number	Latency, s, *M* ± s.e.m.	Short-Term Memory, %	Long-Term Memory, %
Control	8	19.6 ± 7.9	87.5	87.5
HFCD	8	17.0 ± 4.8	62.5	25.0 *
HFCD + Tyr	8	25.5 ± 9.2	25 *	25.0 *
HFCD + Trp	8	7.3 ± 1.6 ***	12.5 *^,^**	0 *^,^**

* Difference with the Control group is significant, *p <* 0.05, Fisher’s exact test. ** Difference with the HFCD group is significant, *p <* 0.05, Fisher’s exact test. *** Difference with the HFCD group, at the trend level, *p* = 0.074, Mann-Whitney *U*-test.

## Data Availability

Data available on request due to restrictions eg privacy or ethical.
